# An open source ultrasonic anemometer for the spatially distributed and time synchronized measurement of large scale flow fields

**DOI:** 10.1016/j.ohx.2026.e00781

**Published:** 2026-04-30

**Authors:** Till S. Weise, Johannes N. Braukmann, Philipp Hartmann

**Affiliations:** a German Aerospace Center (DLR), Institute of Aerodynamics and Flow Technology, Bunsenstraße 10, 37073 Göttingen, Germany; b University of Applied Sciences, Department of Aerospace and Automotive Engineering, Hohenstaufenallee 6, 52064 Aachen, Germany

**Keywords:** Ultrasonic sensor, Flow measurement, Anemometry, Velocimetry, Low cost, Open source

## Abstract

The quantification of flow speed and -direction is key to many aerodynamic investigations. In case of large scale flow fields and outdoor environments optical techniques such as particle image velocimetry or Shake-the-Box are difficult to implement. Instead, a sufficient amount of point-wise measurements can be employed. Ultrasonic anemometers are a compelling solution due to their high accuracy and low drift even at low to moderate flow velocities. Commercial ultrasonic anemometers often lack a synchronization method and an analog output and most of the available products are closed source and therefore the algorithms employed have unknown characteristics such as time delay, error handling and filtering. The goal of this paper is to develop an ultrasonic anemometer that can measure flow velocities up to 35 m s^−1^ and address the above issues while being low cost. The sensor is based on off-the-shelf electronic components, two custom printed circuit boards, and uses an STM32F4 microcontroller as its main processor. A semi-automated calibration and validation process was performed, achieving a mean flow speed magnitude accuracy of ±0.3m/s and an angle error of less than 1%.

## Graphical abstract

**Figure:**
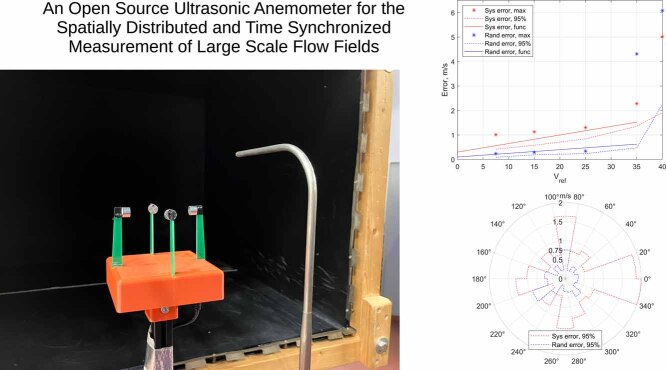

**Table:** 

**Specifications table**	

**Hardware name**	open USAN (UltraSonic ANemometer)

**Subject area**	• Engineering and material science

**Hardware type**	• Field measurements and sensors

**Closest commercial analog**	• Thies 2D ultrasonic anemometer

**Open source license**	Software: BSD-3-Clause and Hardware: CERN-OHL-P-2.0

**Cost of hardware**	Approx. 250€

**Source file repository**	https://doi.org/10.5281/zenodo.19187845

## 1 Hardware in context

Velocity is a key parameter in flow investigations and sufficient measurement techniques have been subject to development for centuries. Different approaches are used to resolve flow fields with a suitable temporal and spatial resolution. Point-wise techniques such as pressure probes, hot wire anemometers, mechanical devices, namely cup- or propeller anemometers as well as high-end planar or even volumetric optical techniques are only a few viable options. All methods feature benefits and drawbacks and are useful in different scenarios.

The majority of heat and momentum based approaches to velocimetry show a significant reduction in accuracy at very low inflow speeds [1], [2]. Hot wires, for example, have excellent frequency response but exhibit considerable drift over time and poor accuracy at low velocities. This is due to the low forced convection at small wind speeds. Cup anemometers have a low overall drift, but due to the mechanical inertia of the rotor, the frequency response is limited and can only be used to measure the magnitude of a 2D flow.

An alternative are ultrasonic anemometers, which are well known from meteorology. Unlike momentum and heat based approaches to velocity measurement, they are exceptionally accurate at measuring very low velocities, feature no moving parts and provide the local magnitude and direction of even 3D flows. In case of a large-scale flow investigation in outdoor conditions and an expected velocity range between 0 and 35m/s with a measurement frequency of up to 100Hz ultrasonic anemometers are a promising choice. Though lower measurement frequencies (<20Hz) are usually sufficient for engineering applications, a higher measurement frequency can be used to resolve smaller length scales or to increase accuracy through oversampling.

The spatial resolution of large-scale measurements is directly affected by the final price per unit whereas for small scale experiments also the sensor size and its blockage are important parameters. Commercially available ultrasonic sensors are often expensive and can be quite large, since they are designed to provide reliable long-term measurements in a wide range of weather conditions. Another disadvantage of commercially available sensors is the lack of precise control over data acquisition timing and synchronization. While velocity can often be read out synchronously, clock mismatch causes the timing of the data acquisition to vary across sensors. Which limits the feasibility of these sensors to be used in a spatially distributed array, where a highly accurate timing is required. Regarding the measured quantities, commercial sensor manufacturers claim accuracies of ±0.1m/s for wind speeds below 10m/s [3] or ±0.2m/s for wind speeds below 5m/s [4]. Due to the closed-source nature of the commercial offerings, not much is known about the inner workings, potential filtering, and resulting delays. Furthermore, no detailed error statistics are available.

For the intended measurement and characterization of helicopter downwash, a 2D measurement often suffices because, in the vicinity of the ground, the flow field flattens and can be assumed to be quasi-2D. The aim of this project is to create an open source 2D ultrasonic anemometer with competitive accuracy to the commercial options, but with lower unit costs. The self-imposed size limit is 100mm×100mm×100mm. The final sensor consists of two 3D printed parts and two PCB designs, one being the main board with the STM32F4 microcontroller and the other being the transducer arm. Due to the outdoor application, care was taken to make the sensor resistant against dripping water for a short time.

The algorithms used and the resulting measurement delay are well documented and openly available to fulfill the open source philosophy.

After a semi-automatic calibration in a wind-tunnel an analysis of the resulting errors is performed. The calibration is performed by calculating and applying a lookup-table. An mean flow magnitude error of ±0.3m/s and a 95% credibility interval of ±1.43m/s for inflow speeds up to 35m/s.

## 2 Hardware description

### 2.1 Measurement principle

**Fig. 1:**
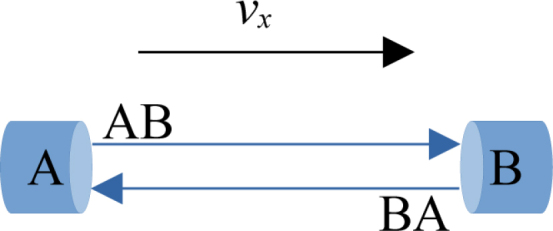
Schematic structure of a 1D ultrasonic anemometer.

The measurement is based on the propagation of sound through the moving medium. If a difference in travel time is measured between two identical signals sent in opposite directions, it can be concluded that the medium in which the sound waves traveled has moved. The transmitted sound pulses accumulate this shift throughout the duration of the propagation and along the distance traveled. The transmission and reception of ultrasonic pulses are facilitated by the use of transceivers designated A and B, respectively (Fig. 1). Both transceivers span the measurement path $l{}_{\text{0}}$. In an ideal case, the air speed can be calculated using

(1) $$v{}_{\text{AB}}=a+v{}_{\text{air,AB}}=\frac{l_{0}}{t{}_{\text{AB}}},$$

where a describes the speed of sound, and $t{}_{\text{AB}}$ represents the time interval between the emission and reception of a signal also known as time of flight (ToF).

A second measurement in the opposite direction is necessary to also estimate the unknown parameter a. Assuming a constant airspeed during the measurement, the measured speed can be expressed as

(2) $$v_{AB}=a-v_{air,AB}⟺v{}_{\text{air,AB}}=v{}_{\text{AB}}-a=\frac{l_{0}}{t{}_{\text{AB}}}-a.$$

The analogue equation can be derived for the BA direction.

The assumption of steady conditions during the measurements in both directions results in

(3) $$v{}_{\text{air,AB}}=-v{}_{\text{air,BA}}.$$

In practice, small deviations from this theoretical assumption are present. The following formula can be derived and used to determine the velocity [5]:

(4) $$v_{x}=\frac{v{}_{\text{air,AB}}-v{}_{\text{air,BA}}}{2}=\frac{l_{0}}{2}\left(\frac{1}{t{}_{\text{AB}}}-\frac{1}{t{}_{\text{BA}}}\right)$$

The measured velocity is denoted $v_{x}$ to acknowledge the difference between actual and measured air speed.

The speed of sound can be calculated using

(5) $$a=\frac{l_{0}}{\frac{1}{2}\left(t{}_{\text{AB}}+t{}_{\text{BA}}\right)}.$$

Note that the above equations only apply when assuming a constant 1D flow along the measurement path. A measurement error will occur if this criterion is not met.

The time between sending and receiving is measured based on a matched filter approach. A matched filter optimizes the signal-to-noise ratio for a given filter kernel [6]. The matched filter is implemented by cross-correlating a match with the measured signal. The term “match” refers to a pre-recorded signal that has been captured under optimal conditions and no wind.

The temporal shift in the correlation peak indicates the time shift of the recorded signal compared to the match. Consequently, the output of the filter is a relative delay $t{}_{\text{rel}}$ to the matched signal. In contrast, Eq. (5) is based on the absolute time between emission and reception of the signal. Therefore, the delay $t_{0}$ between emission and reception at the opposite transceiver at zero wind is estimated by dividing the distance traveled $l_{0}$ by the speed of sound $a_{0}$. For ease of use the speed of sound can be expressed as a function of the ambient temperature $T_{0}$ and two constants: $R_{L}$, the specific gas constant of air, and κ, the isentropic expansion factor.

(6) $$t{}_{\text{0}}=\frac{l{}_{\text{0}}}{a{}_{\text{0}}}=\frac{l{}_{\text{0}}}{\sqrt{\kappa \cdot R_{L}\cdot T_{0}}}$$

The total delay between transmission and reception during a measurement can be readily determined by adding the delay of the matched signal to the relative delay:

(7) $$t{}_{\text{AB}}=t{}_{\text{0}}+t{}_{\text{AB,rel}}.$$

This approach nullifies any offsets due to latency, because both signals experience the same latency which are thus canceled out. Consequently, the temporal alignment of signal emission and reception must be maintained with precision. Any discrepancy in the timing will inevitably result in a measurement error.

**Fig. 2:**
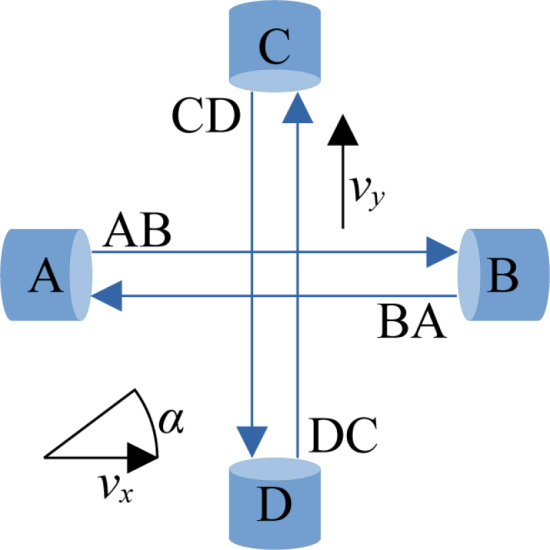
The schematic structure of an 2D ultrasonic anemometer.

For the measurement of 2D air flows, at least two 1D measurements in linear independent orientations are required. The velocities measured along the measurement paths are denoted $v_{x}$ and $v_{y}$ respectively. In this project an orthogonal orientation of both transceiver pairs (as shown in Fig. 2) is used for simplicity and good angle sensitivity. In this configuration the measurement paths intersect at 90° and are located in the same plane. This results in simple trigonometric calculations for the angle α and magnitude $v{}_{\text{mag}}$ of the airflow:

(8) $$\alpha =arctan2\left(\frac{v_{x}}{v_{y}}\right)$$

(9) $$v{}_{\text{mag}}=\sqrt{v_{x}^{2}+v_{y}^{2}}$$

Under real world conditions, errors due to elongation of the travel time caused by cross flow and velocity deficits along the measurement path caused by the blockage of the transceivers create an inflow angle-dependent systematic measurement error. The cross flow leads to an elongation of the travel path of the sound waves. This effect can be approximated by a quadratic function with empirical derived coefficients and the measured velocity in the orthogonal direction. The correction of the velocity deficit caused by blockage and subsequent flow slowdown over the measurement path is more difficult to predict and will be described in more detail in Section 2.3.

### 2.2 Hardware

The sensor is based on an STM32F469 microcontroller, which interfaces with the analog front-ends, trigger, digital-to-analogue converter (DAC), USB, and optionally SRAM. The SRAM can be used to store real-time ADC recordings of up to 400 measurements for later examination. This feature has been used extensively in the development of the sensor. The USB connection is provided by a USB-to-serial bridge IC (FT231XQ-T) which can be used to configure the sensor or for digital data transfer.

The trigger is used to synchronize the sensors and update the DAC. The DAC (AD5732AREZ) has two channels and is used to provide an analogue output signal of the measured x and y velocity components with an output range of ±10  V, which can be reconfigured for other voltage ranges. An external 2.5  V reference (REF3425TIDBVR) is provided for stable and drift-free operation of the DAC. The analogue ground can be separated from the digital ground if required.

The analog front-ends are responsible for amplifying the input and output voltages of the transceiver to the appropriate voltage levels. The piezoelectric transceivers require up to 150  V peak to peak for transmission while the received signal level is less than 200 mV peak to peak.

All inputs and outputs are protected against ESD, over-voltage and reverse polarity. The expected voltage levels are 14  V–24  V from the power supply, an analog output of ±10  V, and a TTL trigger input.

### 2.3 Software

The signal processing can be divided into 3 stages: the matched filter stage, the 1D measurement stage and the 2D measurement stage, which are implemented in the respective functions “Filter_run_r0”, “Filter_Fusion” and “Filter_eval_r0” (the source code can be found in corresponding .c and .h files, located in the Zenodo repository). Each stage reduces the amount of data that is passed on to the next stage. The matched filter converts the raw data into a list of potential delays ($t_{\mathrm{XX}}$), which are then converted into a list of 1D measurements (denoted as $v_{x}$ and $v_{y}$). In a final step, the 1D measurements are combined to produce one 2D measurement ($\left[v_{x},v_{y}\right]$).

The matched filter is implemented using an FFT convolution approach to the correlation [6], the match is transformed once at the initialization of the sensor to reduce the amount of FFTs that need to be performed during each measurement. To reduce the ambiguity of the correlation result, the transmitted signal is encoded using a phase shift keying (PSK) scheme. The least ambiguous key is unique to each transceiver model due to its characteristics. Therefore, if the transceiver model is changed, a new optimum key must be found; this can be done using the key test option, see Section 5.3 for more information.

Due to the mechanical and electrical inertia of the transceiver, no instantaneous phase shift can be performed. The resulting ambiguity, even with the optimum key, is still reasonably high. Especially at high inflow speeds, an increased level of ambiguity is expected. To resolve the problem, more peaks than just the primary correlation peak are examined. In this implementation, the five highest peaks are passed to the next stage.

In the 1D measurement stage, the results of Eqs. (4) and (5) are calculated for the selected peaks of the matched filter. To achieve sub-sample accuracy a quadratic interpolation for each peak is performed. A plausibility analysis is performed on each result based on the resulting speed of sound and the primary peak ratio (PPR) they yield. The three most likely candidates are used for the next step.

The next stage involves an analysis of the difference in measured speed of sound for each pair of 1D measurements and the divergence from long-time average, as well as the PPR. The candidate pair with the highest probability is given as a result. A detailed explanation of the algorithms used can be found in [7].

The extensive plausibility check helps to catch measurement errors caused by ambiguity of the correlation which is more pronounced at high velocities. If no plausible result can be found, an error value is returned, which is later removed from the averaging process by discarding the measurement.

The sending and receiving of the raw signal is offloaded to the microcontroller’s DMA controller, allowing data processing of the previous measurement to take place while the next is being recorded, resulting in an internal measurement frequency of up to 200Hz.

**Fig. 3:**
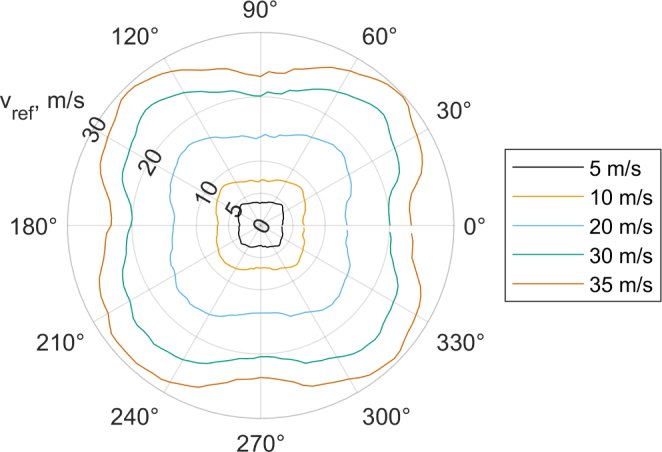
Calibration data.

Fig. 3 is an example of the data collected for calibration (a similar figure is automatically generated when using the calibration script). The polar plot shows the measured absolute velocity on the radial-axis and the inflow angle on the theta-axis. The different colored lines describe the measured velocities at the same inflow magnitude over changing inflow angles. In this case the velocities used for calibration were 5m/s, 10m/s, 20m/s, 30m/s and 35m/s. It can be observed that the measured absolute values resemble more of a square in contrast to the ideal circle. Uncorrected this would lead to a velocity magnitude error exceeding 10m/s and an angle error exceeding 4°.

A major error source is the flow blockage caused by the transceivers. A velocity deficit is induced to the flow, also known as transceiver shadow effect. This deficit is mainly a function of the inflow angle and roughly linear to the inflow velocity, see Fig. 3. Without the blockage the resulting graphs should represent circles at the respective measured speeds. The blockage occurs particularly when a pair of transceivers is in-line with the flow (at 0°, 90°, 180°, and 270°) Traditional compensation methods such as those proposed by Kaimal-Gaynor [8] are not suitable due to the small ratio of transceiver diameter to measurement path length [9]. Therefore, a 2D lookup table is used, which requires a set of calibration measurements to be taken at known inflow angles and velocities. The recording of this calibration can be semi automated using a rotary stage (e.g. the URS75BCC with an SMC100 controller by Newport) and the provided script “Sensor_calibration_and_validation” in the Zenodo repository. This configuration can also be used to validate the sensor.

**Fig. 4:**
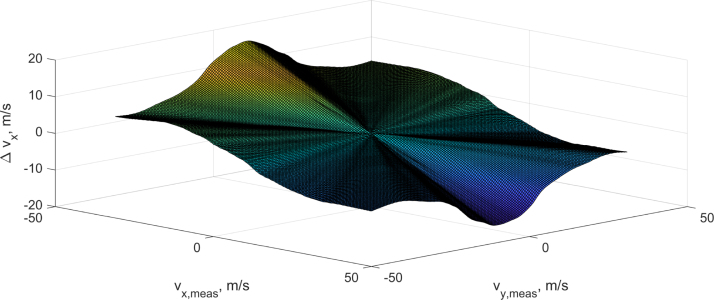
Velocity correction as deviation to reference value for $v_{y}$.

Fig. 4 displays the resulting values of the correction matrix for the $v_{y}$ velocity output with the reference values subtracted.^1^ The measured x- and y-velocity components are displayed on the x- and y-axis while the divergence to the reference values is displayed on the z-axis. On the diagonals of the x- and y-axis the deviation is relatively small and uniform. Around the zero point of the x-axis the deviation is larger, this coincides with the angles where the blockage of $v_{y}$ by the transceivers is the largest. The large blockage also leads to a more non-linear behavior of the measured deviation.

### 2.4 Sensor configuration

The sensor can be configured via a USB virtual COM port with a baud rate of 1 Mbaud. Via the COM port the sensor can be configured to suit the desired application: A new zero-measurement can be performed, the use of an external trigger signal can be configured, and the serial output can be set. After changing the settings, the new values are active, but will be reset by restarting the sensor. To keep the changes, the configuration has to be written to the EEPROM of the device, which can also be done via the COM port. External triggering can be enabled or disabled. If enabled, the maximum expected trigger frequency and duty cycle must be set. The sensor performs an amount of sub-measurements with the constant internal frequency of 200Hz that fit the set maximum expected frequency and the chosen duty cycle. If the set frequency is e.g. 40Hz with a duty cycle of 100%, five sub-measurements are performed after each trigger. The average of the sub-measurements is output on the subsequent trigger event and a new set of sub-measurements is started, creating a latency of exactly one trigger period. In case the actual external trigger signal has a lower frequency, the sensor waits for the next trigger after the precalculated amount of sub-measurements. Effectively this leads to a reduced duty cycle. If the actual external trigger has a higher frequency than the one configured, no reliable data is put out.

If the external triggering is disabled, a measurement frequency and duty cycle can be chosen via the COM port. As in the case with external triggering, the set frequency is filled with internal sub-measurements at 200Hz according to the chosen duty cycle. In contrast to the external trigger case, the data is put out as soon as the measurement is performed.

Data is output via the COM port and the analog output. The maximum possible output rate is 100Hz, while the internal measurement frequency is always 200Hz, the collected sub-measurements are averaged and an outlier detection is applied to reduce the statistical error of the output measurement. If there are no sub-measurements considered reliable, a zero value is output. The implementation only allows integer-divided measurement frequencies of the internal measurement frequency. A good compromise between statistical error and measurement frequency is at around 40Hz with the resulting 5 sub-measurements.

**Table 1:** Overview of the openUSAN design files.

Design filename	File type	Open source license	Location of the file
USAN_Assembly.step	CAD	CERN-OHL-P-2.0	https://doi.org/10.5281/zenodo.19187845
Mainbody.step	CAD	CERN-OHL-P-2.0	https://doi.org/10.5281/zenodo.19187845
Lid.step	CAD	CERN-OHL-P-2.0	https://doi.org/10.5281/zenodo.19187845
USAN_r0	Schematics	CERN-OHL-P-2.0	https://doi.org/10.5281/zenodo.19187845
USAN_TR_r0	Schematics	CERN-OHL-P-2.0	https://doi.org/10.5281/zenodo.19187845
2D-Anemometer-Firmware_r0	Software	BSD-3-Clause	https://doi.org/10.5281/zenodo.19187845

## 3 Design files summary

**Fig. 5:**
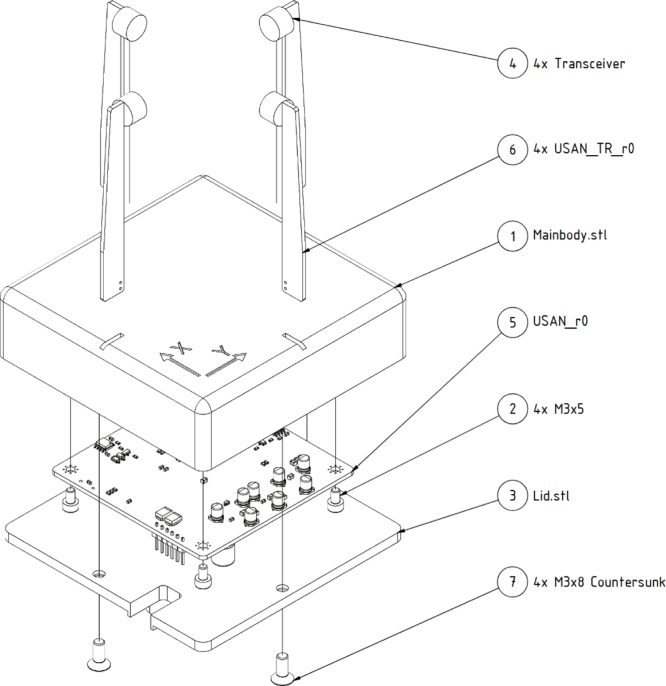
Exploded-view drawing of the USAN.

USAN_Assembly is the main CAD assembly of the sensor and is displayed in Fig. 5. It contains all the required 3D prints and the CAD of the PCBs (see Table 1). The main body and lid are 3D printed parts, both are also available as stl-files in the Zenodo repository. It is recommended to print the parts in ASA if the device is used in UV light. The cover has an adapter to 20mm×20mm aluminum extrusion for mounting, but can be modified for other mounting options. USAN_r0 and USAN_TR_r0 are the KiCAD projects for the mainboard- and transceiver arm PCBs respectively. USAN_r0 also contains the BOM and production files. The PCB will be considered as a finished assembly in the following. 2D-Anemometer-Firmware_r0 is the Segger software solution project used to program the microcontroller. A precompiled version of the code can also be found in the repository. The bill of materials is displayed in Table 2.

**Table 2:** Bill of materials with estimated costs.

Designator	Component	Number	Cost per unit	Total cost
Mainbody.stp	Mainbody	1	0.75€	0.75€

M3 × 5 (DIN 912/ISO 4762)	Screw	4	0.05€	0.20€

Lid.stp	Lid	1	0.62€	0.62€

M3 × 8 (DIN 7991/ISO 10642)	Lid	4	0.05€	0.20€

CUSA-TR60-02-2000-TH67 (Same Sky)	Transducer	4	7.21€	28.84€

USAN_r0	Mainboard	1	160.00€	160.00€

USAN_TR_r0	Transducer arm	4	3.50€	14.00€

Glue (Cyanoacrylate)	Glue	1	0.20€	0.20€

RG178 (MIL-C 17F)	Coax wire	1.5m	2.42€	3.36€

BNC socket	Coax connector	3	3.49€	10.47€

Cable $2\times 0.34\;{\mathrm{mm}}^{2}$	Power wire	0.6m	0.51€	0.31€

AMP Superseal (TE Connectivity 282080-1)	2-pin power connector	1	1.60€	1.60€

Solder wire	Wire	1	0.20€	0.20€

(optional) Solder aide	Solder aide	1	0.07€	0.07€

## 4 Bill of materials summary

Note: The cost given for PCB and cables is dependent of manufacturer and order volume.

An ST-Link programmer (e.g. STLINK-V3MINI) with an 4 pole Pico-Clasp Cable as well as a power supply are needed to flash the microcontroller.

## 5 Build instructions

### 5.1 Preparation

• Check the PCBs for manufacturing defects.
• Solder the power cable and the three coaxial cables to the mainboard, and equip all cables with their respective plugs.
• Solder the transceivers to the USAN_TR_r0 PCB, optionally the soldering aid can be used to create a more repeatable soldering process.
• Check the slots of the mainbody for debris or excessive stringing, if the USAN_TR_r0 PCB fits it is good enough, otherwise clean the slots.
• Check that the lid and mainbody fit together, use sandpaper if necessary.

### 5.2 Assembly

**Fig. 6:**
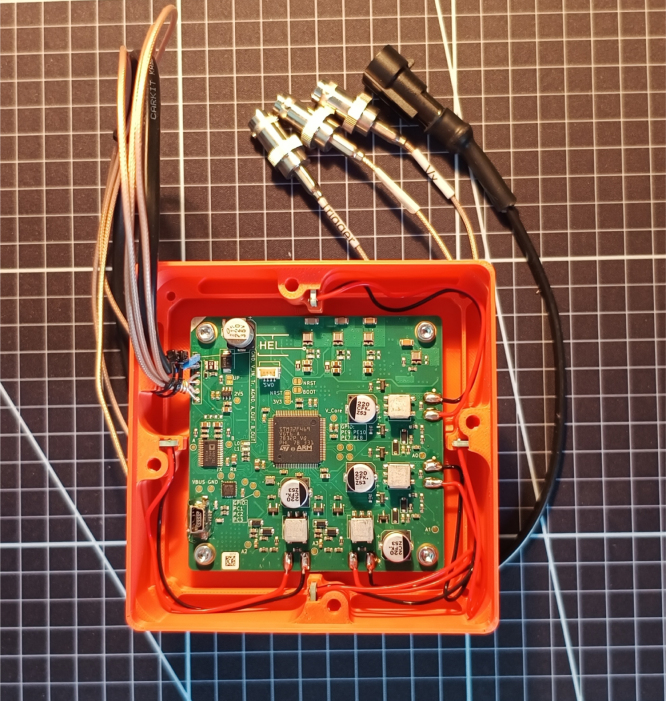
Soldered assembly.

• Insert the USAN_TR_r0 PCBs into the slots with the transceivers facing each other.
• If the fit is loose or a watertight seal is desired, add glue around the edge where transceiver arms and mainbody meet.
• Insert the mainboard into the mainbody, making sure that the white triangle in the corner of the PCB is aligned with the triangular corner of the 3D print and secure the PCB using the M3 × 5 screws.
• Secure the IO cables with zip-ties against the mainbody.
• Connect the solder pads of the USAN_TR_r0 PCB with the pads of the mainboard (compare Fig. 6). For this connection the RG178 coax cabel can be used.
• After flashing the controller use the M3 × 8 screws to secure the lid to the mainbody.

**Fig. 7:**
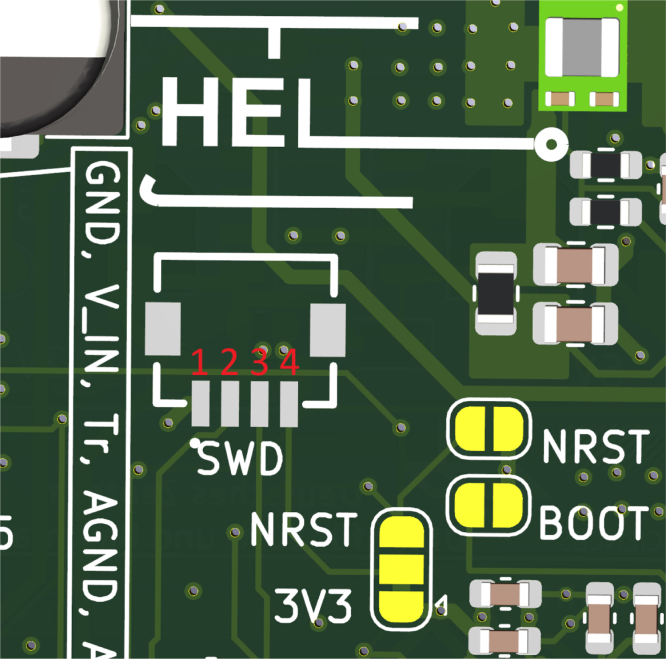
SWD Pinout: 1:GND, 2:CLK, 3:DIO, 4:3V3.

### 5.3 Flashing

To flash the controller, first connect the programmer (ST-Link) to the PCB, the pin-out of the connector is shown in Fig. 7. Flashing can be done in two different ways, either the pre-compiled binary file can be flashed using the STM32CubeProgrammer, or the code can be compiled and flashed using SEGGER Embedded Studio (recommended). When compiling from source, there are some more customization options available in “Sensor_config.h”, which are useful when modifying the sensor, the options are:

• Altering the transceiver distance.
• Adjusting the outlier detection threshold.
• Adjusting the sub-measurement frequency.
• Activating the key test mode.

In order for the matched filter to work, a match has to be pre-recorded. The match must be recorded under no wind conditions. To ensure the no wind condition the sensor should be placed in a cardboard box or similar with at least 20  cm space from each transceiver to the next wall of the box (this includes top and bottom) to avoid artifacts due to reflection. For a useful recording of a match, the ambient temperature has to be known and entered during recording.

Power up the sensor and connect it to the COM port using a Mini-USB cable. The syntax for the COM port to record the match is “-n float(Temperature)”. The “-w” command can be used to save the match to flash (otherwise the match will be lost when the sensor is switched off).

### 5.4 Calibration

The calibration can either be a generic calibration provided with the project or new calibration data can be recorded (e.g. in a wind tunnel) and optionally validated afterwards. The generic calibration will not yield the best sensor accuracy. The calibration script provided with the project is using Matlab. An automated rotary stage is recommended to record the calibration. The provided Matlab script is designed to work with an SMC100 motor controller and a URS75BCC rotary stage, both from Newport (using a 3D printed adapter from rotary stage to the aluminum strut, the part is called “URS_75_mount” and can be found in the Zenodo repository). A high accuracy anemometer is required to determine the inflow velocity, the use of a Prandtl tube in conjunction with a Betz manometer is recommended. The inflow speeds used for calibration are: 5m/s, 10m/s, 20m/s, 30m/s and 35  m/s.

Prior to the calibration connect the COM port of the sensor to a PC using a Mini-USB cable. In case a rotary stage is used, connect it to the PC as well. The Matlab script will guide the user through all the necessary steps for the calibration. First, the two COM ports of the sensor and the rotary stage are required. The script performs a homing of the rotary stage. Afterwards, the script will suggest suitable inflow velocities and wait for the user to enter the actual inflow velocity measured by the reference anemometer. At each wind speed the rotary stage performs a 360° rotation in steps of 3°. After recording the calibration measurements, the lookup table is calculated and a report figure is generated, this step takes approximately 2 min depending on the host PC. The script asks for confirmation if the calibration may be sent and saved onto the sensor, overwriting the old calibration. Finally, a validation can be performed in a subsequent step to validate the calibration at desired wind speeds and randomly chosen inflow angles.

## 6 Operation instructions

The sensor emits high sound pressure audio signals in an audible ultrasonic frequency range, it is advised not to expose the ear for prolonged periods, although the emitted 90  dB SPL sound pressure is well below the 110  dB SPL limit in many international guidelines [10]. Special attention should be given to the exposed transceiver pads due to the potential high voltages ($150\;V_{\mathrm{pp}}$). If used outdoors, the exposed transceiver pads should be coated with a protective coating (e.g. Plasti Dip spray coating).

### 6.1 Setup

The sensor should not be placed close to a wall or large objects. The reflected sound could cause measurement errors. Ideally, the sensor should be oriented with a pair of transceivers in the direction of the flow, as this is the region with the lowest statistical error (see Fig. 11).

To configure the sensor, connect the COM port to the PC with a baud rate of 1  Mbaud. The trigger configuration has the syntax “-t float(trigger frequency in Hz) float(measurement duty cycle in %)”. If the selected trigger frequency is zero, the trigger is disabled, otherwise the measurement will only start when a trigger signal is received. The duty cycle describes how long the sensor will measure in the measurement period, if the period is set to 1, then the sensor will go into double shot mode and record two samples on each trigger event. If trigger is disabled the second argument can be used to control the output frequency. The minimum achievable measurement frequency is 1Hz, the maximum frequency is 100Hz, while the recommended frequency is 40Hz. The resulting number of sub-measurements recorded during one measurement period is returned via the COM port.

The “-a int(mode)” command can be used to configure the ASCII output via the COM port, the options being 0 for no output, 1 for continuous output and 2 for one-time ASCII output.

The currently active setting can be read with the “-s” command, the setting can be permanently saved to the EEPROM with the “-w” command. The “-s” command also states if the currently active settings are the same as the ones stored in the EEPROM.

When the sensor is first powered up, the two analog outputs will generate a square wave of five periods (−9.0  V, 0.0  V, +9.0  V) with a period length of 1  s each. This signal can be used to check for a good connection to the external data acquisition system and to compensate for offsets and non-linearity.

### 6.2 Measurement

The sensor is tested in air speeds of up to 40m/s, all speeds above this level may result in higher measurement error, the implemented absolute limit is 50m/s. The analog output has a scaling factor of $5\frac{\text{m/s}}{\text{V}}$ which means that 9  V correspond to a wind speed of 45m/s. The 2D-velocity is output as velocity components $v_{x}$ and $v_{y}$.

If the external trigger is enabled, the result of the measurement will be output on the next trigger event, resulting in an output delay of one trigger period. This is done to have a repeatable and well characterized timing characteristic. If the trigger signal is recorded in addition to the analog output signals, it can be used to easily extract regions of valid data.

## 7 Validation and characterization

This section evaluates the overall expected error and presents an easy-to-use error estimation.

**Fig. 8:**
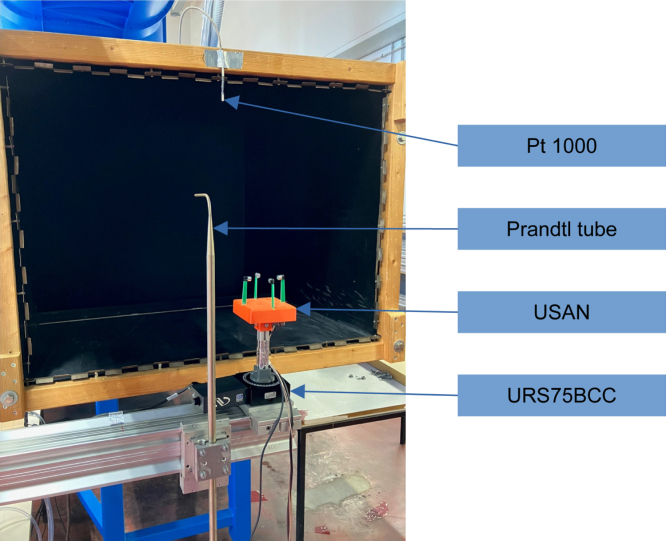
Validation and Calibration setup.

The validation of the sensor was carried out in the 1-meter wind tunnel (1MG) at the German Aerospace Center (DLR) in Göttingen. For the determination of the reference speeds a Prandtl tube in combination with a Betz manometer was used, the angle adjustment was performed by a URS75BCC rotation stage with an SMC100 controller both by Newport. The measurement setup is shown in Fig. 8. All components were mounted on an aluminum truss frame and placed in front of the nozzle in the open test section. The size of the nozzle was 1m×0.7m (width × height). The validation setup differs from the calibration setup in terms of the reference sensors used and the climate conditions.

The utilized validation script (“Only_validation” in the Zenodo repository) automatically creates test points at user defined speeds and a user defined number of random inflow angles. The script communicates with the motor controller and reads the sensor data via the COM port.

For the characterization of the accuracy, data of four different USAN sensors were collected at inflow speeds of 7.5m/s, 15m/s, 25m/s, 35m/s and 40m/s, at each speed 50 random angles were tested. Resulting in 1000 measurement points in total. All tested inflow speeds, except at the inflow speed of 35m/s, differ from the speeds used for calibration. For each point 100 measurement samples were taken at a sampling rate of 40Hz. Based on the fixed frequency of the sub-measurements of 200Hz, five sub-measurements are performed per measurement sample. This data set will be used as the basis for the measurement error evaluation.

A classical approach to error description will be taken:

$$v=\bar{v}+\epsilon$$

where v represents the measured speed at each measurement sample of a measurement point, $\bar{v}$ describes the mean measured speed of all 100 measurement samples at the measurement point, and ϵ the mean free residuum, which represents the random error. The standard deviation is used as the metric for the random error. The systematic error $E_{\mathrm{sys}}$ will be defined as the absolute difference of the reference airspeed $v_{\mathrm{ref}}$ and mean measured speed $\bar{v}$.

$$E_{\mathrm{sys}}=\left|v_{\mathrm{ref}}-\bar{v}\right|$$

**Fig. 9:**
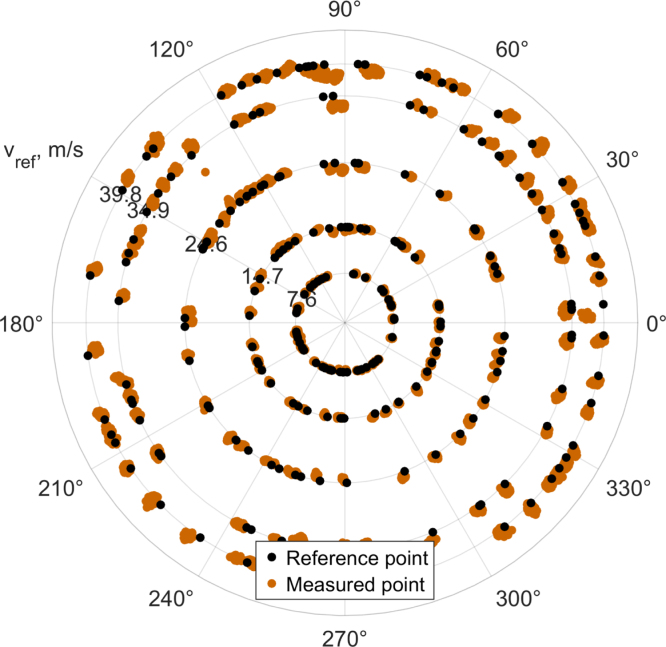
Sample data of a characterization.

Fig. 9 shows the measured points recorded with one sensor. The polar plot displays the reference points as black dots and the measured data of the sensor in ocher.

An overall systematic angle error of about 2° was observed, one possible explanation of this error would be a misalignment error between the calibration and validation setup. The angle error will not be characterized in further detail since the overall error is small. The observed systematic inflow angle error typically declined with higher inflow speeds. This can be ascribed to the conditioning of the conversion from x- and y- velocities to magnitude and angle, compare Eq. (8).

The systematic velocity magnitude error is noticeably larger at 0°, 90°, 180° and 270°, when the transceiver blockage is the largest. Part of this might be attributed to the change in air temperature between calibration, performed in summer and validation performed in winter, which could lead to different magnitudes of velocity deficit behind the transceivers.

**Fig. 10:**
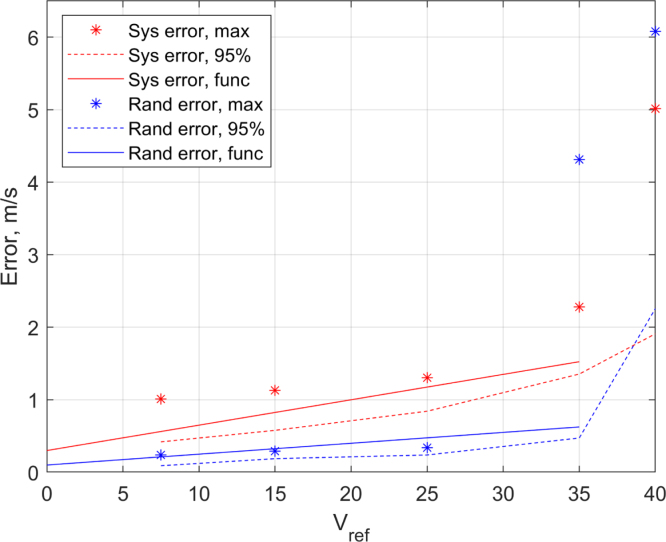
Error over inflow speed.

Fig. 10 shows the systematic error (red) and random error (blue). Both error trends are plotted over the reference velocity magnitude on the x-axis. The maximum deviation is depicted by asterisks, while the 95% percentile is displayed with a dotted line. Solid lines represent the proposed error estimation functions. All measurement errors rise approximately linear with the inflow speeds. At speeds above 35m/s the errors rise disproportionately strong resulting in a maximum systematic error of over 5m/s, and a random error exceeding 6m/s standard deviation.

**Fig. 11:**
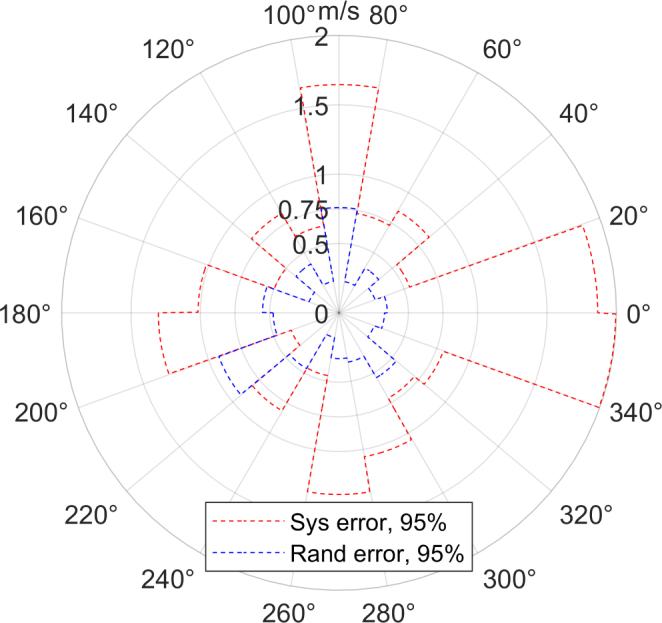
Error over inflow angle.

Fig. 11 displays the magnitude of the 95% percentile of the random (blue) and systematic (red) measurement error on the radial axis and the inflow angle on the angular axis. All measurements are binned in 20° inflow angle bins over all sensors and inflow velocities. The systematic error is notably larger around the inflow angles of 0°, 90°, 180° and 270°, reaching a peak deviation of over 2m/s. The maximum standard deviation occurs in the 200° to 220° bin with a magnitude of 0.92m/s. For maximum precision the sensor should be placed in a 45° angle to the expected inflow.

For further characterization a Kolmogorov–Smirnov-Test^2^ (K-S test) was performed on the data with a rejection level of α=5%. As the reference distribution a Gaussian distribution with the according mean and standard deviation of each point was selected. On less than 10% of all points the null hypothesis was rejected, rejections mostly occurred at 40m/s at angles where one of the transceiver pairs was in line with the inflow. Thus, the statistical error can be assumed to be Gaussian distributed.

The resulting overall error, for inflow speeds below 35m/s can be described as follows for the 95% credibility interval. For the velocity magnitude the standard deviation can be approximated by the following equations:

$$s_{v_{\mathrm{mag}},95\text{\%}}\leq 0.1\;\mathrm{m/s}+0.015\cdot v_{meas,mag}$$

and

$$E_{v_{\mathrm{mag}},95\text{\%}}\leq 0.2\;\mathrm{m/s}+0.035\cdot v_{meas,mag}$$

for the systematic error.

For the angle measurement the standard deviation can be described as

$$s_{\mathrm{angle},95\text{\%}}\leq \text{0.7°}$$

while the mean error can be estimated as:

$$E_{\mathrm{angle},95\text{\%}}\leq \text{3.6°}$$

These estimates hold true for the case of five sub-measurements, the standard deviation can be scaled to other numbers of sub-measurements by multiplying with an expansion factor k which can be calculated by

$$k=\frac{\sqrt{5}}{\sqrt{n}}$$

where n describes the new number of sub-measurements. Because many aerodynamic properties depend on temperature and gas compensation, significant deviations from the calibration conditions may result in larger errors.

## 8 Conclusion and outlook

A 2D ultrasonic anemometer with a measurement range of up to 35m/s and a maximum measurement rate of 100Hz was developed, calibrated, validated, and characterized. The sensor consists of four transceivers in an orthogonal configuration and a main PCB. The main steps in the signal acquisition and processing were examined. The build and configuration options are shown. The sensor was calibrated and validated in a semi-automatic process and a characterization of the residual measurement error was performed. The sensor achieved mean accuracy of ±0.3m/s for inflow speeds below 35m/s. The measurement exhibits a systematic inflow angle dependent error, a correction method was chosen and examined. While the accuracy is in the range of that of commercial offerings, the error is well characterized, and the measurement delay and the internal processing steps are known due to the open source approach of the project.

The achieved material costs of the sensor are below 250  € even when produced in a small batch of 20 units.

As a next step, the calibration and correction procedure will be examined in more detail, with the goal of further reducing the error of the measurement. Furthermore, a 3D version of the sensor can be derived from the current design.

## CRediT authorship contribution statement

**Till S. Weise:** Writing – original draft, Software, Methodology, Data curation. **Johannes N. Braukmann:** Writing – review & editing, Validation, Supervision, Investigation, Data curation. **Philipp Hartmann:** Writing – review & editing, Software, Resources, Methodology, Data curation, Conceptualization.

## Declaration of competing interest

The authors declare that they have no known competing financial interests or personal relationships that could have appeared to influence the work reported in this paper.

## Data Availability

All design files are accessible at Zenodo via the following link: https://doi.org/10.5281/zenodo.19187845.

## Footnotes

1 Note: The subtraction is only performed for visualization purposes and not part of the correction matrix.

2 The Kolmogorov–Smirnov test is a hypothesis test, were the Null hypothesis is, that a given sample is from a given reference distribution.
