# Corrigendum to “An open source ultrasonic anemometer for the spatially distributed and time synchronized measurement of large scale flow fields” [HardwareX 26 (2026) 781]

**DOI:** 10.1016/j.ohx.2026.e00829

**Published:** 2026-08-19

**Authors:** Till S. Weise, Johannes N. Braukmann, Philipp Hartmann

**Affiliations:** a German Aerospace Center (DLR), Institute of Aerodynamics and Flow Technology, Bunsenstraße 10, 37073 Göttingen, Germany; b University of Applied Sciences, Department of Aerospace and Automotive Engineering, Hohenstaufenallee 6, 52064 Aachen, Germany

The authors regret that, in the originally published version of this article, an incorrect Zenodo repository DOI was provided throughout the article.

The DOI:

https://doi.org/10.5281/zenodo.19187845

should read:

https://doi.org/10.5281/zenodo.20144324

This correction applies to all occurrences of the Zenodo repository DOI throughout the article.

The authors would like to apologize for any inconvenience caused.

